# Protective Effects of Leonurine on Alcoholic Liver Injury Through Modulation of Oxidative Stress and JAK2-STAT3 Signaling

**DOI:** 10.3390/cimb48040372

**Published:** 2026-04-02

**Authors:** Shen-Sheng Xiao, Pin-Pin Liu, Hang Zhu, Xue-Dong Wang, Wen-Ping Ding, Xin Liu, Min Fang, Ke-Jia Wu, Zhi-Yong Gong

**Affiliations:** 1 Key Laboratory for Deep Processing of Major Grain and Oil, Ministry of Education, Wuhan Polytechnic University, Wuhan 430023, China; xsswhpu730@163.com (S.-S.X.); liupinpin0724@outlook.com (P.-P.L.);; 2 Hubei Key Laboratory for Processing and Transformation of Agricultural Products, Wuhan Polytechnic University, Wuhan 430023, China; 3 Wuxi School of Medicine, Jiangnan University, Wuxi 214082, China

**Keywords:** Leonurine, alcoholic liver, JAK2, STAT3, network pharmacology, metabolomics

## Abstract

Alcoholic liver disease (ALD) is a prevalent and progressive hepatic disorder driven by chronic excessive alcohol consumption. Leonurine (LH), a bioactive alkaloid isolated from *Herba Leonuri*, possesses well-documented antioxidant and cytoprotective properties. This study comprehensively investigated the hepatoprotective efficacy of LH against ethanol-induced liver injury and mechanistically dissected its molecular underpinnings. Antioxidant capacity and cytoprotective activity were assessed in ethanol-treated hepatocytes. Network pharmacology and gene expression analysis were performed to identify potential therapeutic targets and signaling pathways. UHPLC–MS/MS-based metabolomics was applied to characterize endogenous metabolic alterations induced by LH. LH demonstrated significant antioxidant activity and was predicted to interact with 44 ALD-related targets. Functional enrichment and gene validation analyses revealed that its protective effects were primarily associated with regulation of the JAK2–STAT3 signaling pathway. Metabolomic profiling identified 48 differential metabolites and 25 significantly affected metabolic pathways. Integrated analysis of metabolites and target genes further supported the JAK–STAT signaling pathway as a central regulatory axis, which was confirmed in cellular experiments. Collectively, these results demonstrate that LH confers hepatoprotection in ALD primarily through modulation of the JAK2–STAT3 signaling pathway, underscoring its translational promise as a mechanism-informed therapeutic candidate.

## 1. Introduction

Leonurus japonicus belongs to the Labiatae family that grows in Asia, the temperate regions of Europe and Africa [1]. Leonurine (LH), a bioactive alkaloid isolated from Herba Leonuri, has garnered substantial scientific interest due to its well-documented antioxidant properties—particularly its capacity to ameliorate mitochondrial and cellular dysfunction, suppress intracellular reactive oxygen species (ROS) accumulation, and restore redox homeostasis [2,3,4]. Mechanistically, LH enhances AKT phosphorylation, thereby inhibiting caspase activation and modulating the Bcl-2/Bax ratio to attenuate oxidative stress-induced apoptosis. A growing body of evidence indicates that LH-mediated mitigation of mitochondrial impairment and potentiation of endogenous antioxidant defenses collectively contribute to the attenuation of pathogenic progression in multiple acute inflammatory disorders [5,6].

Chronic excessive alcohol consumption induces hepatic oxidative stress and may progress to alcoholic hepatitis or cirrhosis [7]. Alcoholic liver disease (ALD) is a spectrum of alcohol-associated hepatic disorders ranging from steatosis to fibrosis and end-stage cirrhosis, directly attributable to prolonged heavy alcohol intake. The pathogenesis of ALD is fundamentally driven by alcohol-induced oxidative damage, which disrupts redox homeostasis and promotes hepatocellular injury. Specifically, ethanol metabolism elevates the hepatic NADH/NAD^+^ ratio through the sequential oxidation of ethanol to acetaldehyde (by alcohol dehydrogenase) and subsequently to acetate (by aldehyde dehydrogenase), resulting in mitochondrial dysfunction, impaired fatty acid oxidation, and accumulation of reactive oxygen species (ROS) [7,8,9,10,11]. Our prior work demonstrated that LH ameliorates ethanol-induced hepatotoxicity by concurrently modulating oxidative stress responses and glycerophospholipid metabolism [12]. Nevertheless, the precise molecular targets and signaling pathways mediating LH’s therapeutic effects in ALD remain incompletely characterized.

The JAK2–STAT3 signaling pathway plays a pivotal role in regulating oxidative stress, inflammatory responses, and hepatocyte survival. Its dysregulation is strongly associated with the pathogenesis and progression of alcohol-induced liver injury, whereas targeted modulation of this pathway has demonstrated therapeutic potential in mitigating hepatocellular damage [13]. In this study, we evaluated the hepatoprotective effects of LH against ALD by assessing its antioxidant capacity, cytoprotective activity. Network pharmacology was employed to identify the potential targets and molecular mechanisms of LH against ALD. Metabolisms and genes evaluation were applied to study the changes of endogenous substances induced by LH in ethanol-induced liver cells. We suggested that LH is a promising hepatoprotective antioxidant against ALD through modulation of the JAK2–STAT3 signaling pathway.

## 2. Materials and Methods

### 2.1. Reagents

Roswell Park Memorial Institute (RPMI) 1640 medium and fatal bovine serum (FBS) were purchased from Gibco by Thermo Fisher Scientific Inc. (Shanghai, China). Penicillin–streptomycin solution was obtained from Genom (Hangzhou, China). DMSO was obtained from Sigma-Aldrich Inc. (Shanghai, China). The hepatocyte cells (LO2 cells) were obtained from Chinese Academy of Science (Cell Biology of Shanghai Institute, Shanghai, China). Leonurine (LH) and ethanol (ET) were purchased from TargetMol Chemicals Inc. (Shanghai, China).

### 2.2. Cell Culture and Treatment

LO2 cell line was cultivated in 1640 medium with 1% penicillin/streptomycin and 10% FBS at 37 °C in a humidified atmosphere (95%) containing 5% CO_2_. LO2 cells were treated with 300 mM ET for 24 h, followed by exposure to LH (10 μM) or JAK2 inhibitor V (10 μM, Proteintech, Cat#CM09350) for an additional 24 h prior to analysis.

### 2.3. Hepatoprotective Efficacy of LH Against ALD

#### 2.3.1. MTT Assay

LO2 cells were seeded at 5000 cells/well in a 96-well plate and incubated overnight at 37 °C. To evaluate the cytotoxicity of ET, cells were treated with increasing concentrations of ET (0–1 M) for 24 h, followed by an MTT assay to determine cell viability [14,15]. For the assessment of protective effects, cells were exposed to ET (300 mM) for 24 h and subsequently treated with LH (0–50 μM) for an additional 24 h, with at least six replicates per group. After treatment, MTT solution (0.5 mg/mL) was added to each well and incubated for 4 h at 37 °C. The culture medium was then carefully removed and replaced with 100 μL of DMSO to dissolve the formazan crystals. Following 10 min of shaking at room temperature in the dark, absorbance was measured at 490 nm using a microplate reader to determine cell viability.

#### 2.3.2. Antioxidant Ability of LH

To evaluate the antioxidant ability of LH, reactive oxygen species (ROS) and superoxide dismutase (SOD) level were evaluated by ROS and SOD detection kits (Nanjing Jiancheng Bioengineering Institute, Nanjing, China). The experiment was conducted in accordance with the operating procedures provided by the manufacturer. Specifically, 2.5 × 10^5^ cells were seeded into 6-well plates. After exposing ET (300 mM) to LO2 cells for 24 h, the cells were treated with LH (0–50 μM) for an additional 24 h. The cells were subsequently collected and washed twice with ice-cold phosphate-buffered saline (PBS). The samples of each experimental group (with a minimum of three replicates per group) were processed following the manufacturer-provided user guide and analyzed immediately using a Multi-Mode Microplate Reader.

### 2.4. Predicting the Mechanism of LH for the Treatment of ALD by Using a Bioinformatics Approach

#### 2.4.1. Prediction of the Potential Targets of LH Against ALD

The potential targets of LH were predicted using SwissTargetPrediction (https://www.molecular-modelling.ch/swiss-drug-design.html accessed on 15 January 2026) and SuperPred 3.0 (https://prediction.charite.de/index.php accessed on 15 January 2026). The targets of ALD were retrieved from the GeneCards database v5.26.0 (http://www.genecards.org/). All targets were restricted to “Homo sapiens” and converted to their official gene symbols. The collected target genes were then input into the Venn diagram tool (Venny 2.1) (https://bioinfogp.cnb.csic.es/tools/venny/ accessed on 15 January 2026) to identify the overlapping genes between LH and ALD for subsequent analysis.

#### 2.4.2. The Potential Mechanism of LH Against ALD

The obtained potential therapeutic targets were then entered into STRING 12.0 (https://string-db.org/) for PPI network analysis. Meanwhile, pathway information for these targets was retrieved from the Kyoto Encyclopedia of Genes and Genomes (KEGG) database (https://www.kegg.jp/), with the species restricted to Homo sapiens for KEGG pathway identification. Enrichment analyses for KEGG and Gene Ontology (GO) were performed using the R clusterProfiler software package(R X64 4.1.2).

### 2.5. Western Blot Analysis

Total cellular proteins were extracted according to a previously described protocol [16,17]. Equal amounts of protein (30 µg per sample) were separated by 10% SDS–PAGE and subsequently transferred onto PVDF membranes (Millipore) at a constant current of 300 mA for 1 h. Membranes were blocked with 5% skim milk (BD Pharminigen, Cat#232100, Franklin Lakes, NJ, USA) prepared in TBST (0.1% Tween-20) for 2 h at room temperature to prevent nonspecific binding. The membranes were then incubated with the appropriate primary antibodies (Affinity Biosciences, Cat#AF6022, Cat#AF3022, Cat#AF6294, Cat#AF3293) overnight at 4 °C, followed by three washes with TBST (10 min each). After washing, membranes were incubated with the corresponding secondary antibodies for 3 h at room temperature. Immunoreactive bands were detected using an enhanced chemiluminescence (ECL) kit (Vazyme, Cat#E422-02) and visualized with a Tanon 4600SF imaging system (Shanghai, China). Band intensities were quantified using ImageJ software (Version 1.54r).

### 2.6. HPLC-MS/MS Metabolomics (Quasi-Targeted Metabolomics)

#### 2.6.1. Collection and Preparation of Samples

A total of 2.5 × 10^6^ cells were seeded into 6-well plate. Following exposure of LO2 cells to ET (300 mM) for 24 h, the cells were treated with LH (10 μM) for an additional 24 h. After collection, the cells underwent two washing cycles using ice-cold PBS. Cell samples were transferred to microcentrifuge tubes and resuspended in 80% methanol by vortex mixing. The samples were then thawed on ice and vortexed for 30 s under stable conditions. Following 6 min of sonication, the samples were centrifuged at 5000 rpm for 1 min at 4 °C. The supernatant was freeze-dried and reconstituted in 10% methanol. Finally, the solution was injected into the HPLC-MS/MS system for analysis.

#### 2.6.2. HPLC-MS/MS Analysis

HPLC-MS/MS analyses were performed using an ExionLC™ AD system (SCIEX) coupled with a QTRAP^®^ 6500+ mass spectrometer (SCIEX) at Novogene Co., Ltd. (Beijing, China). Samples were injected onto an HSS T3 column (2.1 × 150 mm, 2.5 μm) and separated using a 20 min linear gradient at a flow rate of 0.4 mL/min under both positive and negative ion mode. Mobile phase A consisted of 0.1% Formic acid–water, and mobile phase B consisted of 0.1% Formic acid-acetonitrile [18]. QTRAP^®^ 6500+ mass spectrometer was operated in positive ion mode with Curtain Gas of 35 psi, Collision Gas of Medium, Ion Spray Voltage of 5500 V, Temperature of 550 °C, Ion Source Gas of 1:60, Ion Source Gas of 2:60. QTRAP^®^ 6500+ mass spectrometer was operated in negative ion mode with Ion Spray Voltage of −4500 V, and other parameters remained consistent [19].

#### 2.6.3. Metabolites Identification and Quantification

The samples were analyzed based on the Novogene in-house database (Novogene Bioinformatics Technology Co., Ltd., Beijing, China) using the Multiple Reaction Monitoring (MRM). The Q3 (daughter ion) were used to the metabolite quantification. The Q1 (parent ion), Q3 (daughter ion), retention time (RT), declustering potential (DP), and collision energy (CE) were used to the metabolite identification. The HPLC-MS/MS-generated data files were analyzed using the SCIEX OS software (Version 1.4) to perform peak integration and correction [19].

#### 2.6.4. Metabolic Pathways Analysis

Raw metabolomics data were processed according to our previously reported protocol. Briefly, peak alignment, retention time correction, and normalization were performed prior to statistical analysis. All metabolomics experiments were conducted using four independent biological replicates per group (*n* = 4) [20]. To explore global metabolic differences among groups, principal component analysis (PCA) was first applied as an unsupervised multivariate method to visualize clustering trends and detect outliers. Subsequently, orthogonal partial least squares–discriminant analysis (OPLS-DA) was performed as a supervised method to maximize intergroup discrimination. Model reliability was evaluated using R^2^ and Q^2^ parameters and validated by permutation testing to avoid model overfitting [18]. Variable importance in projection (VIP) scores derived from the OPLS-DA model were used to estimate the contribution of each metabolite to group separation. Differential metabolites were identified based on the following criteria: VIP > 1.0, FDR-adjusted *p* < 0.05 (Benjamini–Hochberg correction), and fold change (FC) > 2 or <0.5. For hierarchical clustering analysis, intensity values of differential metabolites were normalized using z-scores and visualized using the heatmap package in R software (R X64 4.1.2). Functional annotation and pathway enrichment analysis were conducted using the Kyoto Encyclopedia of Genes and Genomes (KEGG) database. Metabolic pathway impact and enrichment analyses were performed using MetaboAnalyst 5.0 (www.metaboanalyst.ca) [19].

### 2.7. Statistical Analysis

All experiments were performed with at least three independent biological replicates (*n* > 3). Data are presented as mean ± SEM. Statistical analyses were conducted using GraphPad Prism 5.0. For comparisons among multiple groups, one-way analysis of variance (ANOVA) was applied, followed by Dunnett’s post hoc test for comparisons against the control group. A *p*-value of < 0.05 was considered statistically significant. For metabolomics data, Student’s t-test was used for pairwise comparisons in combination with multivariate analysis (PCA and OPLS-DA) as described above.

## 3. Results

### 3.1. Identification of LH as an Antioxidant Agent Against Alcoholic Liver Injury

**Figure 1:**
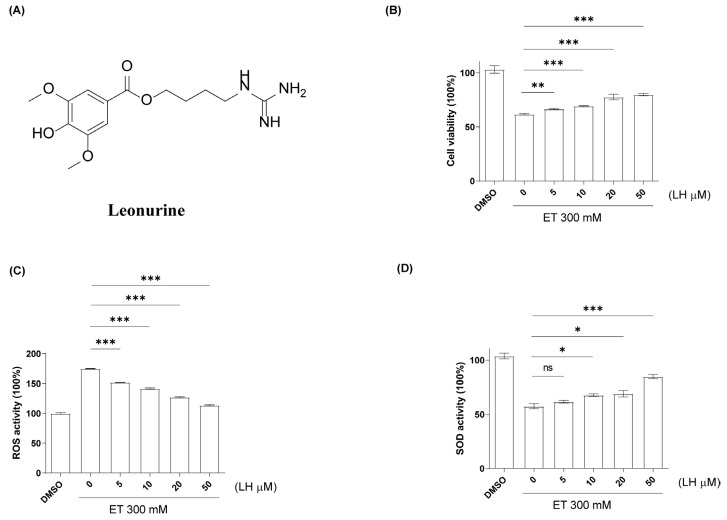
The antioxidative activity of LH (0–50 μM) against ethanol-treated LO2 cells. (**A**) The structure of LH. (**B**) The cell viability of ethanol-treated LO2 cells after exposing to LH (0–50 μM) for 24 h. (**C**) ROS level was decreased in a dose-dependent manner by treatment with LH (0–50 μM) compared to ethanol-treated LO2 cells group. (**D**) SOD level was increased in a dose-dependent manner by treatment with LH (0–50 μM) compared to ethanol-treated LO2 cells group. Data are presented as mean ± SEM (*n* = 6). *, ** and *** denote significant differences at *p* < 0.05, *p* < 0.01 and *p* < 0.001, respectively, between ethanol-treated LO2 cells group and LH-exposed ethanol-treated LO2 cells group. ns denotes no significant difference.

To determine an appropriate ethanol concentration for establishing an in vitro alcoholic liver injury model, LO2 cells were first exposed to increasing concentrations of ET, and cell viability was assessed by MTT assay. The results demonstrated that ET induced a concentration-dependent reduction in cell viability. Among the tested concentrations, 300 mM ET caused a significant but non-lethal decrease in cell viability, thereby providing an adequate injury window for evaluating the protective effects of LH (Figure S1). Therefore, 300 mM ethanol was selected as the modeling concentration for subsequent experiments. To investigate the hepatoprotective effects of LH (Figure 1A), cell viability was assessed after treating ethanol-exposed LO2 cells with LH (0–50 μM) for 24 h. The results revealed that LH significantly attenuated ethanol-induced cytotoxicity in a dose-dependent manner (Figure 1B). To further evaluate the antioxidant action of LH against alcoholic-induced liver oxidative damage, ROS and SOD were evaluated after exposing LH (0–50 μM) to ethanol-treated LO2 cells for 24 h. The results showed that LH can suppress the induction of ROS caused by alcohol-induced oxidative damage and promote the recovery of SOD in liver cells (Figure 1C,D). These findings indicate that LH may alleviate alcohol-induced liver damage through the activation of the antioxidant defense system.

### 3.2. LH Targets and Potential Therapeutic Targets of ALD

**Figure 2:**
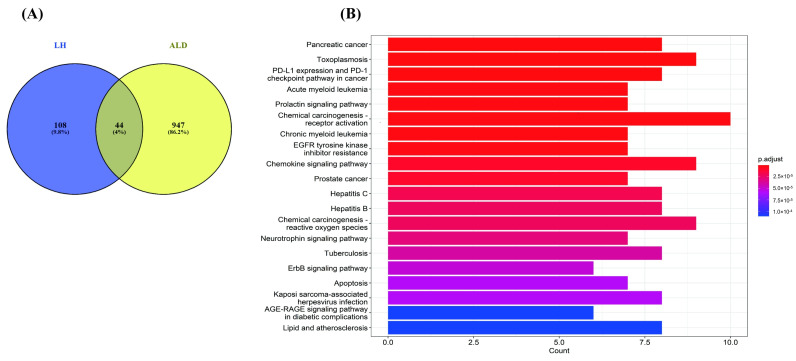
Bioinformatic analyses of LH-ALD intersection proteins. (**A**) Venn diagram of LH targets and ALD proteins. (**B**) KEGG annotation.

To further investigate the potential mechanism of LH against ALD, disease genes were collected from Genecard database. The potential therapeutic targets genes of LH were acquired and deduplicated by Superpred and Swiss database. A total of 949 genes associated with ALD and 108 potential target genes of LH were identified. Then, the Venn diagram viewer extracted a total of 44 common genes to ALD and LH as potential therapeutic targets for the following analysis (Figure 2A).

### 3.3. The Underlying Mechanisms of LH Against ALD

To explore the potential functions of LH on ALD, 44 potential target genes of LH were employed to KEGG pathways enrichment analyses. Top 20 KEGG signaling pathways were obtained and constructed (Figure 2B). Top 6 signaling pathways regulated by LH were Pancreatic cancer (hsa05212, *p*-Value = 3.62 × 10^-9^, Count = 8), Toxoplasmosis (hsa05145, *p*-Value = 3.89 × 10^-9^, Count = 9), PD-L1 expression and PD-1 checkpoint pathway in cancer (hsa05235, *p*-Value = 1.29 × 10^-8^, Count = 8), Acute myeloid leukemia (hsa05221, *p*-Value = 3.98 × 10^-8^, Count = 7), Prolactin signaling pathway (hsa04917, *p*-Value = 5.43 × 10^-8^, Count = 7), Chemical carcinogenesis—receptor activation (hsa05207, *p*-Value = 8.23 × 10^-8^, Count = 10). The enriched GO terms ranked by *p*-value in biological processes, molecular functions, and cell components are presented in Figure S2. Top-three terms in GO biological processes were cellular response to peptide (GO:1901653), response to oxygen levels (GO:0070482), and neuron death (GO:0070997) (Figure S2A). In terms of GO molecular functions, they were mainly enriched in protein serine kinase activity (GO:0106310), protein serine/threonine kinase activity (GO:0004674), and endopeptidase activity (GO:0004175) (Figure S2B). The top-three terms in the GO cellular component category were cell leading edge (GO:0031252), secretory granule lumen (GO:0034774), and cytoplasmic vesicle lumen (GO:0060205) (Figure S2C). After analysis of the underlying mechanism of the 44 potential target genes of LH, a protein–protein interaction (PPI) network was constructed by STRING (Figure S2D). In this network, the central nodes with high degree were ABL1, FOXO1, AKT1, AKT2, STAT3, and mTOR. These findings implied that the central genes may serve as key therapeutic targets of LH in the treatment of ALD.

### 3.4. Metabolite Profiling of ALD Treated with LH

**Figure 3:**
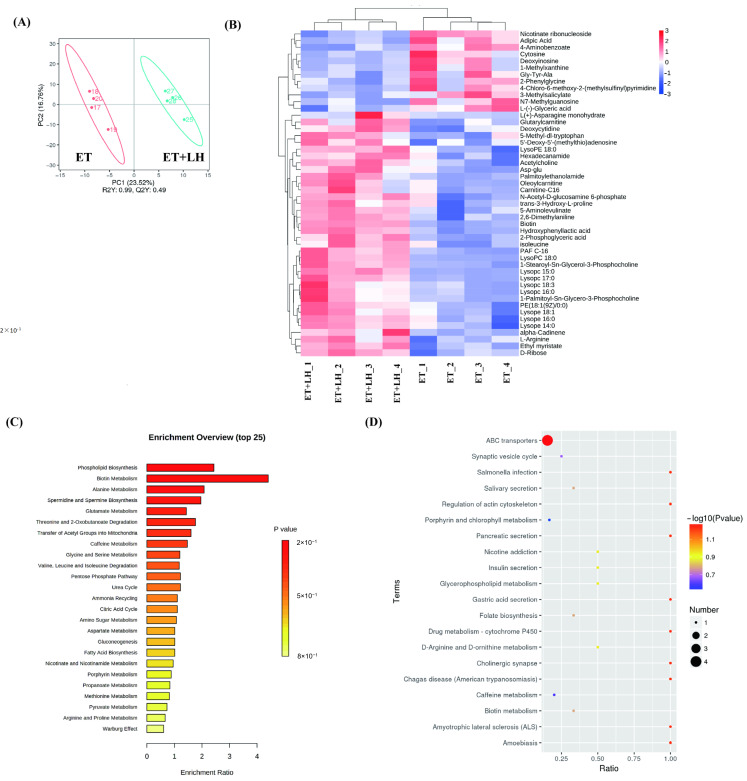
Overall qualitative and quantitative analysis of metabolomics data. (**A**) PCA of metabolite profiling data. (**B**) Identification of differentially accumulated metabolites. (**C**) Metabolite set enrichment analysis. (**D**) KEGG enrichment of different metabolites.

To investigate the metabolite profiling of ALD treated with LH, we subsequently conducted UHPLC-MS/MS analysis to examine the metabolic profile of LO2 cells treated with ET following exposure to LH (10 μM) for 24 h. Differential metabolites between the ET and ET + LH groups were identified through multivariate statistical analyses, including OPLS-DA and PCA, with selection criteria set as VIP > 1, *p* < 0.05, and fold change thresholds of >1.2 or <0.83 [20,21]. These results showed that the ET group, and the ET + LH group exhibited a clear separation trend, indicating that LH intervention had a significantly effect on these metabolites of liver cells exposure to ET (Figure 3A). The clustering heatmap results indicated that there were 48 significantly different metabolites between the ET group and the ET + LH group (Figure 3B). The integral levels of 1-Methylxanthine, Adipic Acid, Deoxyinosine, Nicotinate ribonucleoside, Gly-Tyr-Ala, 4-Aminobenzoate, 4-Chloro-6-methoxy-2-(methylsulfinyl)pyrimidine, 2-Phenylglycine, N7-Methylguanosine, Cytosine, L-(−)-Glyceric acid, and 3-Methylsalicylate were increased in the ET + LH group comparing with ET group. In contrast, expression of Glutarylcarnitine, 5-Aminolevulinate, alpha-Cadinene, trans-3-Hydroxy-L-proline, L-Arginine, N-Acetyl-D-glucosamine 6-phosphate, isoleucine, Palmitoylalcohol amide, Acetylcholine, Deoxycytidine, Ethyl myristate, Asp-glu, Lysope 18:1, 5′-Deoxy-5′-(methylthio)adenosine, Lysope 16:0, Hexadecanamide, Lysope 14:0, D-Ribose, PE(18:1(9Z)/0:0), Lysopc 18:3, 2-Phosphoglyceric acid, LysoPE 18:0, 5-Methyl-dl-tryptophan, 2,6-Dimethylaniline, Carnitine-C16, Lysopc 16:0, Biotin, 1-Palmitoyl-Sn-Glycero-3-Phosphocholine, Oleoylcarnitine, Hydroxyphenyllactic acid, 1-Stearoyl-Sn-Glycerol-3-Phosphocholine, LysoPC 18:0, PAF C-16, Lysopc 17:0, Lysopc 15:0, and L(+)-Asparagine monohydrate were downregulated in the ET + LH group comparing with ET group.

### 3.5. Metabolic Pathway Remodeling Induced by LH in ALD

Differentially expressed metabolites were subjected to pathway enrichment analysis using MetaboAnalyst 5.0, which identified 25 candidate metabolic pathways. Among these metabolites, Acetylcholine and LysoPC(16:0) were involved in the Phospholipid Biosynthesis pathway; Biotin was participated in the Biotin Metabolism pathway; 5′-Methylthioadenosine was associated with Spermidine and Spermine Biosynthesis; L-Arginine and 5-Aminolevulinic acid were related to Glycine and Serine Metabolism; Biotin and N-Acetyl-D-Glucosamine 6-Phosphate were associated with the Glutamate Metabolism; L-Isoleucine and Biotin were associated with the Valine, Leucine and Isoleucine Degradation; 1-Methylxanthine was related to Caffeine Metabolism (Figure 3C). Collectively, these results suggest that LH-induced modulation of ALD-associated metabolic dysregulation involves perturbations in biotin metabolism, phospholipid biosynthesis, and glycine–serine–threonine metabolism. To further investigate the potential mechanistic underpinnings of LH in ALD treatment, integrated KEGG pathway mapping (https://www.kegg.jp/kegg/) was performed. This analysis identified 5-Aminolevulinate, D-Ribose, Biotin, and L-Arginine as the top-ranked differentially regulated metabolites following LH treatment. Notably, the ATP-binding cassette (ABC) transporter pathway emerged as a significantly enriched metabolic pathway, implicating its potential contribution to the hepatoprotective effects of LH against ALD (Figure 3D).

### 3.6. JAK2–STAT3 Signaling Pathway Is Essential for the Hepatoprotective Effects of LH Against ALD

**Figure 4:**
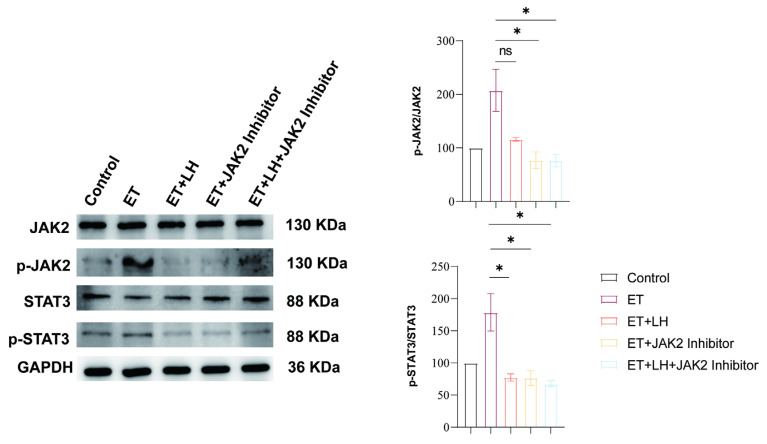
JAK2–STAT3 signaling pathway involvement in the hepatoprotective effects of LH against ethanol-induced liver injury. Representative Western blot images showing total JAK2, total STAT3, phosphorylated JAK2 (p-JAK2), and phosphorylated STAT3 (p-STAT3) in cells. Densitometric analysis of the p-JAK2/JAK2 and p-STAT3/STAT3 ratios is presented. Data are expressed as mean ± SEM from three independent biological replicates (*n*= 3). ns denotes no significant difference, and * denotes significant differences at *p* < 0.05.

To obtain more information on the hepatoprotective effects of LH in addressing alcoholic liver injury, the correlation between the different metabolites and DEGs was analyzed by MetaboAnalyst 5.0. Interestingly, following LH treatment, the JAK–STAT signaling pathway was identified as a central regulatory pathway mediating LH’s protective effects against alcoholic liver injury (Figure S3). This finding is consistent with the network pharmacology analysis, which suggested that STAT3 plays a critical role in the therapeutic effects of LH against ALD. To experimentally validate this prediction, we assessed the protein expression levels of key components in the JAK2–STAT3 signaling pathway. Western blot analysis demonstrated that LH markedly reversed ethanol-induced alterations in the phosphorylation levels of JAK2 and STAT3, with minimal effects on the total protein expression of JAK2 and STAT3. These findings indicate that LH primarily regulates the activation status of the JAK2–STAT3 signaling pathway rather than affecting total protein abundance. To further verify the involvement of JAK2 signaling, a selective JAK2 inhibitor (JAK2 inhibitor V) was applied prior to LH treatment. Notably, pharmacological inhibition of JAK2 abolished the regulatory effects of LH on JAK2 and STAT3 phosphorylation, indicating that the protective action of LH is dependent on JAK2 activity. Together, these results confirm that modulation of the JAK2–STAT3 signaling pathway is essential for the hepatoprotective effects of LH against ethanol-induced injury (Figure 4).

## 4. Discussion and Conclusions

Chronic excessive alcohol consumption induces hepatic oxidative stress and may progress to alcoholic hepatitis or cirrhosis [7]. Leonurine is a bioactive alkaloid isolated from Herba Leonuri and has attracted considerable scientific attention due to its potent antioxidant properties [2,3,4,12]. LH enhances the activities of endogenous antioxidant enzymes—including SOD and glutathione peroxidase (GPx)—and improves mitochondrial function, thereby suppressing mitochondrial ROS generation and restoring adenosine triphosphate (ATP) biosynthesis [22]. In our previous study, we demonstrated that LH exhibits potent antioxidant activity against ALD and identified glycerophospholipid metabolism as a key contributor to its hepatoprotective effects during alcohol-induced oxidative stress [12]. However, the precise molecular mechanisms and therapeutic targets underlying LH’s efficacy in ALD remain incompletely characterized [23,24,25]. In this study, we evaluated the antioxidant and cytoprotective activities of LH against alcoholic liver injury and investigated its underlying mechanism by integrating network pharmacology with UHPLC-MS/MS-based metabolomic analysis. We found that LH exerts therapeutic effects in ALD primarily through amelioration of oxidative damage mediated by 44 putative molecular targets. Among these genes, the central genes including ABL1, FOXO1, AKT1, AKT2, STAT3, and mTOR were identified as key target genes of LH for the treatment of ALD by network pharmacology and PPI network analysis.

Metabonomics analysis can be employed to study the metabolite changes and metabolic pathways in cells [26]. A total of 48 differential endogenous metabolites between ET and ET + LH groups were observed. We found these metabolites were mainly associated with nucleotide and its derivates, phospholipids, and amino acid and its derivatives. By KEGG analysis, ABC transporter metabolic pathway was supposed as the main metabolic pathway of LH for the treatment of ALD. ABCA1 is a key ATP-binding cassette transporter involved in cholesterol efflux and lipid homeostasis in hepatocytes. Upregulation of ABCA1 may enhance the export of cholesterol and phospholipids to apolipoproteins, thereby influencing intracellular lipid composition and downstream metabolic profiles. This mechanism provides a biologically plausible explanation for the observed dysregulation of lipid-related metabolites and the significant enrichment of ABC transporter-associated pathways [27,28]. Collectively, these findings indicate that LH exerts its therapeutic effects in ALD, at least in part, through modulation of hepatic lipid metabolism—thereby supporting lipid metabolic reprogramming as a viable therapeutic strategy for acute liver injury [29]. Notably, prior studies have reported that activation of ABCA1 can influence the JAK2–STAT3 signaling pathway and modulate inflammatory responses [30]. Correlation analysis in the present study also indicated a potential association between metabolic alterations and the JAK–STAT pathway. It is important to emphasize that network pharmacology is a predictive computational strategy that infers target–pathway associations based on database integration and topological algorithms. Such approaches cannot independently establish direct molecular interactions or causal biological relationships. Therefore, the in silico results in this study were used to prioritize candidate signaling pathways for experimental verification rather than to define definitive mechanistic conclusions. Among the predicted pathways, the JAK2–STAT3 axis emerged as a central node based on both network topology and pathway enrichment analyses, and was therefore selected for further validation.

The JAK-STAT signaling pathway is important for many biological functions including proliferation, inflammation, and survival [31,32]. Under physiological conditions, STAT3 is phosphorylated by JAK2, leading to its nuclear translocation and transcriptional activation of target genes [33]. After activated JAK2-STAT3 pathway, STAT3 protein subsequently translocated from cytoplasm into nucleus followed by triggering the target genes to modulate cell proliferation and survival [34,35,36,37]. In this study, Western blot analysis demonstrated that LH significantly reversed ethanol-induced alterations in the phosphorylation levels of JAK2 and STAT3, without markedly affecting total protein expression. Importantly, pharmacological inhibition of JAK2 abrogated the regulatory effects of LH on STAT3 phosphorylation, indicating that JAK2 activity is required for LH-mediated pathway modulation. These data provide direct protein-level evidence supporting the functional involvement of the JAK2–STAT3 axis in LH-mediated hepatoprotection.

In conclusion, this study demonstrates that LH exerts protective effects against ethanol-induced liver injury in vitro. The hepatoprotective activity of LH is mediated through modulation of the JAK2–STAT3 signaling pathway. These findings provide experimental evidence linking LH treatment to regulation of the JAK2–STAT3 signaling pathway in alcoholic liver injury; however, further studies are required to elucidate the precise molecular mechanisms involved.

## Data Availability

The original contributions presented in this study are included in the article/Supplementary Material. Further inquiries can be directed to the corresponding authors.

## Supplementary Materials

The following supporting information can be downloaded at: https://www.mdpi.com/article/10.3390/cimb48040372/s1.
