# Supplementary material for: Protective Effects of Leonurine on Alcoholic Liver Injury Through Modulation of Oxidative Stress and JAK2-STAT3 Signaling
Source: Curr Issues Mol Biol. 2026 Apr 2;48(4):372. doi: 10.3390/cimb48040372 (PMC13114670; doi:10.3390/cimb48040372)
Supplement: Supplementary file 1 [file cimb-48-00372-s001.zip › cimb-4129405-supplementary.pdf]

Supplementary Material

Protective Effects of Leonurine on Alcoholic Liver Injury Through Modulation of Oxidative Stress and JAK2-STAT3 Signaling

Shen-Sheng Xiao [1,2†], Pin-Pin Liu [1,2†], Hang Zhu [1,2] ,Xue-Dong Wang[1,2], Wen-Ping Ding [1,2], Xin Liu [1,2], Min Fang [1,2], Ke-Jia Wu [3,*], and Zhi-Yong Gong [1,2,*]

[1] Key Laboratory for Deep Processing of Major Grain and Oil of Ministry of Education, Wuhan Polytechnic University, Wuhan 430023, China

[2] Hubei Key Laboratory for Processing and Transformation of Agricultural Products, Wuhan Polytechnic University, Wuhan 430023, China

[3] Wuxi School of Medicine, Jiangnan University, Wuxi 214082, China

[†] These authors contributed equally to this work

* Correspondence:
Kejia Wu
kj-wu@outlook.com

Zhiyong Gong
gongzycn@whpu.edu.cn

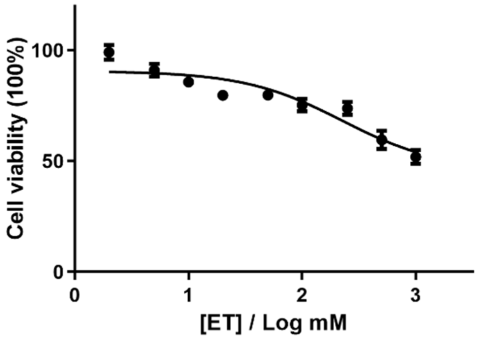

Figure S1. Cell viability of ET against liver cells. LO2 cells were treated with ET (0-1000 mM) for 24h, cell viability was evaluated by MTT assay.

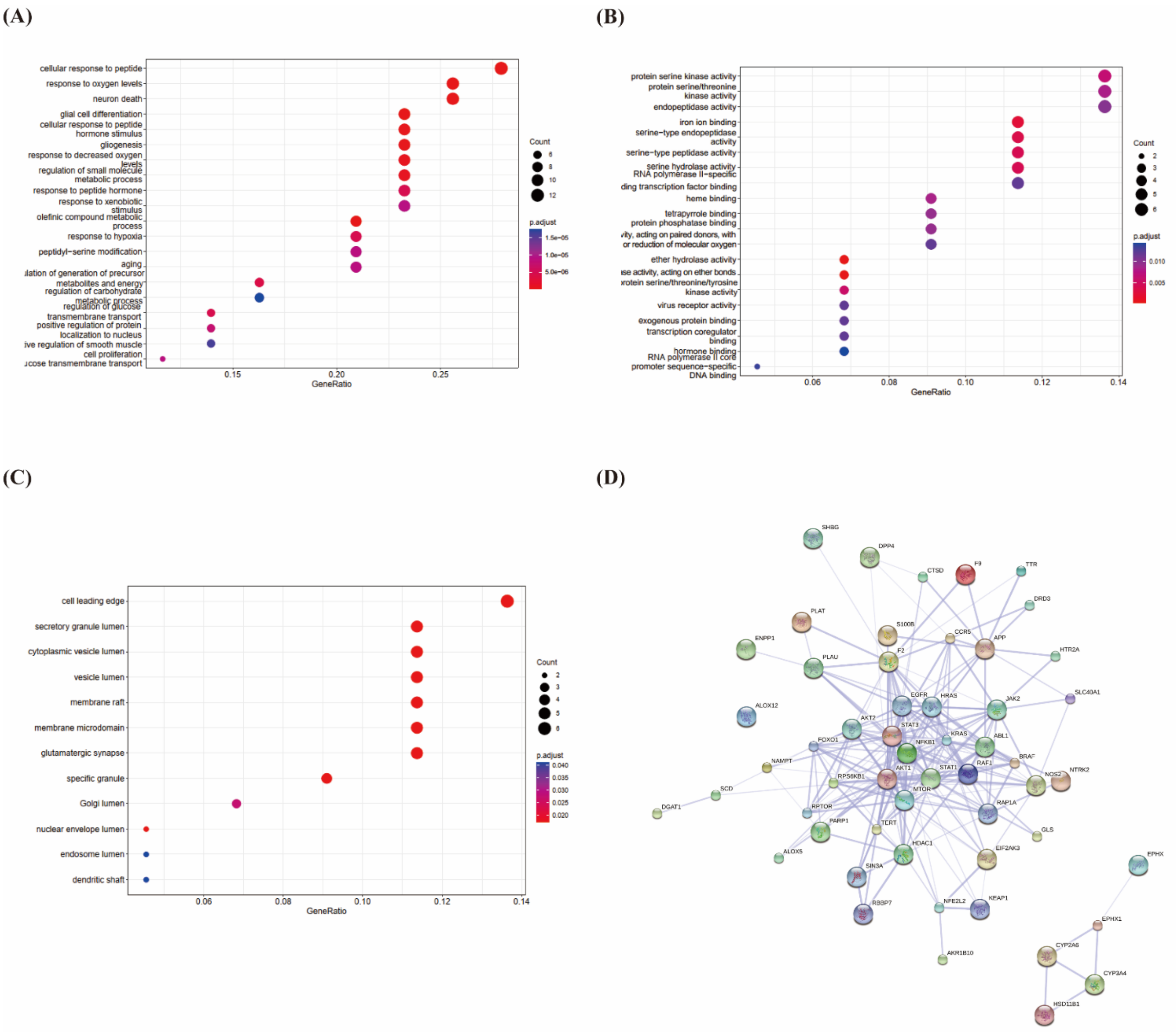

Figure S2. (A) Gene Ontology (GO) enrichment analysis. Different colors represent different gene ontology: Biological processes (BP). (B) Gene Ontology (GO) enrichment analysis. Different colors represent different gene ontology: Molecular functions (MF). (C) Gene Ontology (GO) enrichment analysis. Different colors represent different gene ontology: Cellular component (CC). (D) Protein-protein interaction network of LH acting on ALD. Small circles: protein of unknown 3D structure; large circles: some 3D structure is known or predicted.

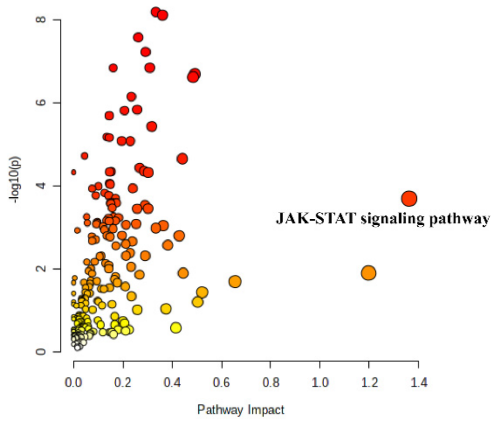

Figure S3. (A) Metabolic pathway analysis by MetaboAnalyst 5.0. Pathway impact value based on the pathway topology analysis. The x-axis represents the pathway impact value computed from pathway topological analysis, and the y-axis is the-log of the p-value obtained from pathway enrichment analysis.
